# Identification of Potential miRNA-mRNA Regulatory Network in the Development of Oral Cancer

**DOI:** 10.1155/2022/9376608

**Published:** 2022-08-18

**Authors:** Yun Yang, Xin Xin, Ting Xu

**Affiliations:** ^1^Department of Stomatology, The Affiliated Yantai Yuhuangding Hospital of Qingdao University, Yantai 264000, China; ^2^Department of Stomatology, North China Medical and Health Group XingTai General Hospital, Xingtai 054000, China; ^3^Department of Stomatology, The Affiliated Yantai Yuhuangding Hospital of Qingdao University Laishan Branch, Yantai 264000, China

## Abstract

**Background:**

Oral cancer is a difficult question in modern medical system, and there are few effective strategies to completely heal these diseases. This research investigated the miRNA-mRNA network in oral cancer development via bioinformatics excavation.

**Methods:**

GSE28100 and GSE23558 in the GEO database were selected for bioinformatics analysis. The datasets were analyzed with GEO2R to obtain the related matrix files. The hot plot and heatmap of the matrix files were drawn with R language. The MiRDIP database was applied to predict and screen the targets of miRNAs. The DEGs in the matrix files were analyzed with the DAVID database and visualized with R language for enrichment analysis. The PPI-network of the DEGs was established with the STRING database and Cytoscape. Besides, the miRNA-mRNA was visualized by Cytoscape.

**Results:**

35 genes were identified as the DEGs in GES28100. 1651 genes were identified as the DEGs in GSE23558. 143 common genes in the targets of miRNAs in GSE28100 and the DEGs in GSE the targets of DEGs in GES28100 and common genes were enriched in the PI3K/AKT pathway, MAPK pathway, etc. The DEGs in GSE28100 and GSE23558 were involved in the regulations of transcription from RNA polymerase II promoter and DNA transcription. The DEGs in GSE28100 and GSE23558 were established with the miRNA-mRNA network.

**Conclusion:**

This research identified miR-15b-5p, miR-199a-3p, miR-21-5p, miR-424-5p, and miR-454-3p as the biomarker of oral cancer and established the miRNA-mRNA network in oral progression.

## 1. Introduction

Oral cancer is a widely prevalent disease in the world, which is one of intractable issues for model society [1, 2]. At present, surgical operation, chemotherapy, and radiotherapy are major therapeutic strategies for oral cancer. However, even with the various clinical intervention, the prognosis of the patients remains unsatisfactory [3]. Statistically, less 50% patients with oral cancer have survival regions more than five years [4]. Increasing studies have indicated that the mRNA profiles of the oral cancer tissues popularly exhibit significant difference compared with the normal tissues [5]. For one side, mRNA disorder is closely related with the deterioration of the cancer, and some genes also exhibit extremely carcinogenic activity to drive the malignant phenotype of the tumor cells [6]. The oral cancer is characterized with a high risk of lymphatic metastasis, which is the major reason leading the rapid progression and worse prognosis of the symptom [7, 8].

miRNAs serve critical roles in cellular metabolism, and the disorder of miRNA profile has also been confirmed as the direct reason causing the development and progression of multiple diseases [9]. For oral cancer, accumulating researches have revealed that the development of this disease is also closely associated with the cellular metabolic disturbance mediated by aberrant expression of some miRNAs [8, 10]. Bioinformatics analysis based on public data of microarray analysis has been gradually recognized as a useful way to investigate potential molecular relationship in disease development [11]. Abundant researches are concentrated on identifying potential drug targets and revealing complex cellular conduction via analyzing the global gene profiles [10]. For oral cancer, an obvious difference of miRNA and mRNA profile is also observed by abundant reports [12]. Some genes may have great value in clinical diagnosis and treatment.

In this study, the potential regulation of oral cancer was investigated via excavating the public cancer database, in order to identify the biomarkers in the development of oral cancer and thus provide some new reference for the treatment of this disease.

## 2. Materials and Method

### 2.1. Data Source

GSE28100 and GSE23558 were obtained from the GEO database (https://www.ncbi.nlm.nih.gov/geo/), and the datasets were analyzed with GEO2R to obtain matrix profiles. GSE28100 was the miRNA expression matrix based on the GPL10850 platform, which included 17 oral cancer tissues and 3 normal tissues. GSE23558 was the mRNA expression matrix based on the GPL6480 platform, which included 27 oral cancer tissues and 5 normal tissues.

### 2.2. Differential Expression Genes (DEGs)

The raw data of GSE28100 and GSE23558 were analyzed with GEO2R tool, and the related matrix files were normalized with the R language. The genes in GSE28100 and GSE23558 with |LogFC| ≥ 2 and *P* < 0.05 were selected as the DEGs.

### 2.3. Function and Pathway Enrichment Analysis

The annotation and enrichment analysis of the DEGs was performed by DAVID database (https://david.ncifcrf.gov/home.jsp). The GO term and KEGG pathways with *P* < 0.05 in the results were screened and then visualized with R language.

### 2.4. Identification of the Hub Nodes

The protein interaction network was analyzed with the STRING database (https://cn.string-db.org/). After that, the results were analyzed and figured with Cytoscape software. For the miRNA-mRNA network, the related miRNAs with differential expression and their targets were figured with Cytoscape software.

## 3. Results

### 3.1. Identification of DEGs

**Figure 1:**
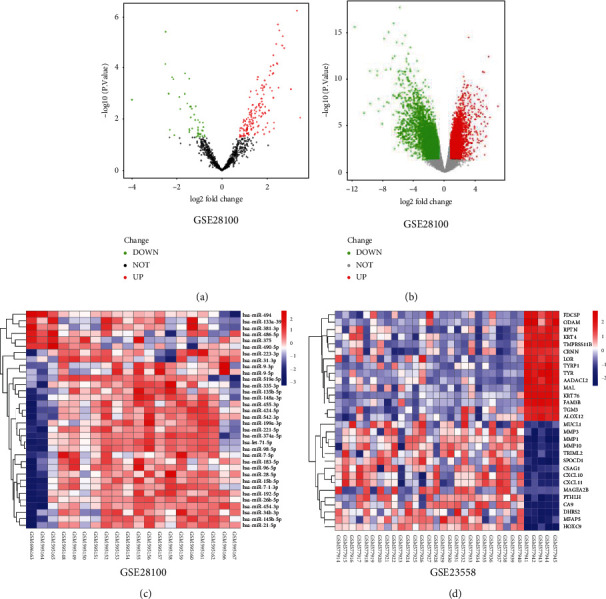
The DEGs in GES28100 and GSE23558. (a, b) The hot plots of DEGs in GES28100 and GSE23558. (c, d) The heatmaps of DEGs in GES28100 and GSE23558.

For GES28100, the abundances of 7 downregulated genes and 28 upregulated genes were identified as the DEGs. For GSE23558, the 1237 downregulated genes and 414 upregulated genes were identified as the DEGs (Figures 1(b) and 1(d)). Moreover, there were 143 common genes in the targets of miRNAs in GSE28100 and the DEGs in GSE (Figures 1(a) and 1(c)).

### 3.2. KEGG Enrichment Analysis

**Figure 2:**
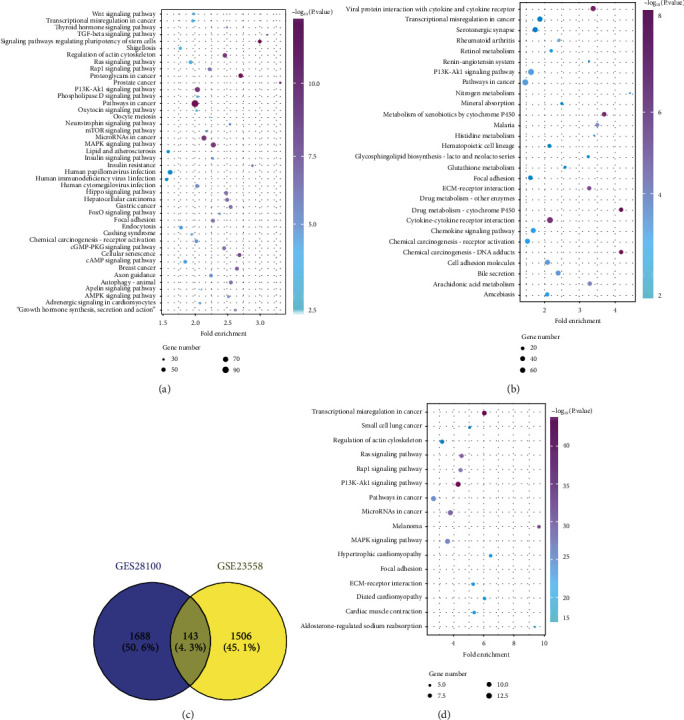
The related KEGG pathways of the DEGs. (a) The KEGG analysis of the DEGs in GES28100. (b) The KEGG analysis of the DEGs in GSE23558. (c) The common genes of GES28100 and GSE23558. (d) The KEGG analysis of the common genes.

The genes were uploaded on the DAVID database to analyze the related pathways. The results showed that targets of DEGs in GES28100 were associated with PI3K/AKT, MAPK, Wnt, Hippo, and APMK pathways (Figure 2(b)). The DEGs in GSE23558 were also related with the PI3K/AKT, MAPK, Wnt, Hippo, and APMK pathway (Figure 2(c)). Moreover, the common genes were enriched in the PI3K/AKT, MAPK pathway, and so on (Figures 2(a) and 2(d)).

### 3.3. GO Enrichment Analysis

**Figure 3:**
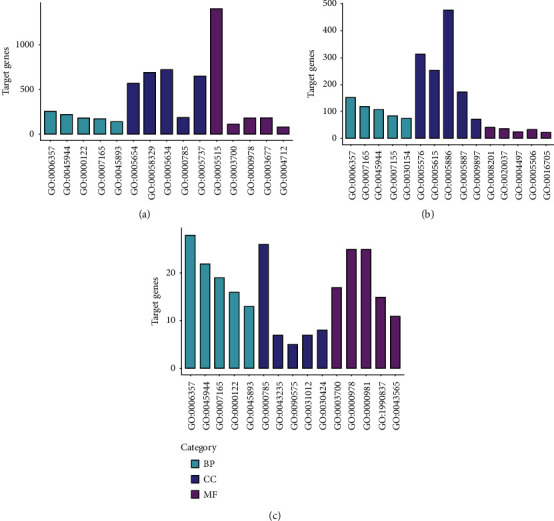
The GO analysis of the DEGs. (a) The GO analysis of the DEGs in GES28100. (b) The GO analysis of the DEGs in GSE23558. (c) The GO analysis of the common genes.

For GSE28100, the molecular functions of the targets were majorly located in nucleus, cytosol, cytoplasm, nucleoplasm, and plasma membrane, and they were related with protein binding, metal ion binding, ATP binding, identical protein binding, and RNA binding and involved in biological processes including the regulation of transcription from RNA polymerase II promoter, signal transduction, and the regulation of DNA transcription (Figure 3(a)). For GSE23558, the DEGs were majorly located in protein binding, plasma membrane, integral component of membrane, extracellular region, and identical protein binding, and they were related with the extracellular space, regulation of RNA polymerase II, and signal transduction and involved in the exosome secretion, integral component of plasma membrane, calcium ion binding, and endoplasmic reticulum membrane (Figure 3(b)). Moreover, the common genes were located in nucleus chromatin, extracellular region, extracellular space, and perinuclear region of cytoplasm, and they were involved in regulation of RNA polymerase II and signal transduction (Figure 3(c)).

### 3.4. PPI and miRNA-mRNA Network

**Figure 4:**
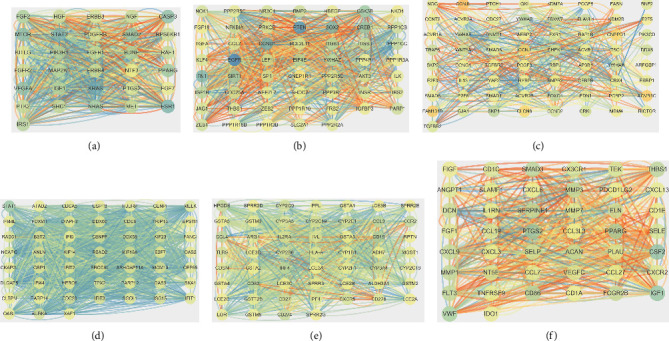
The PPI network of the DEGs in GES28100 and GES23558. (a–c) The PPI network of the DEGs in GES28100. (d, e) The PPI network of the DEGs in GES23558.

**Figure 5:**
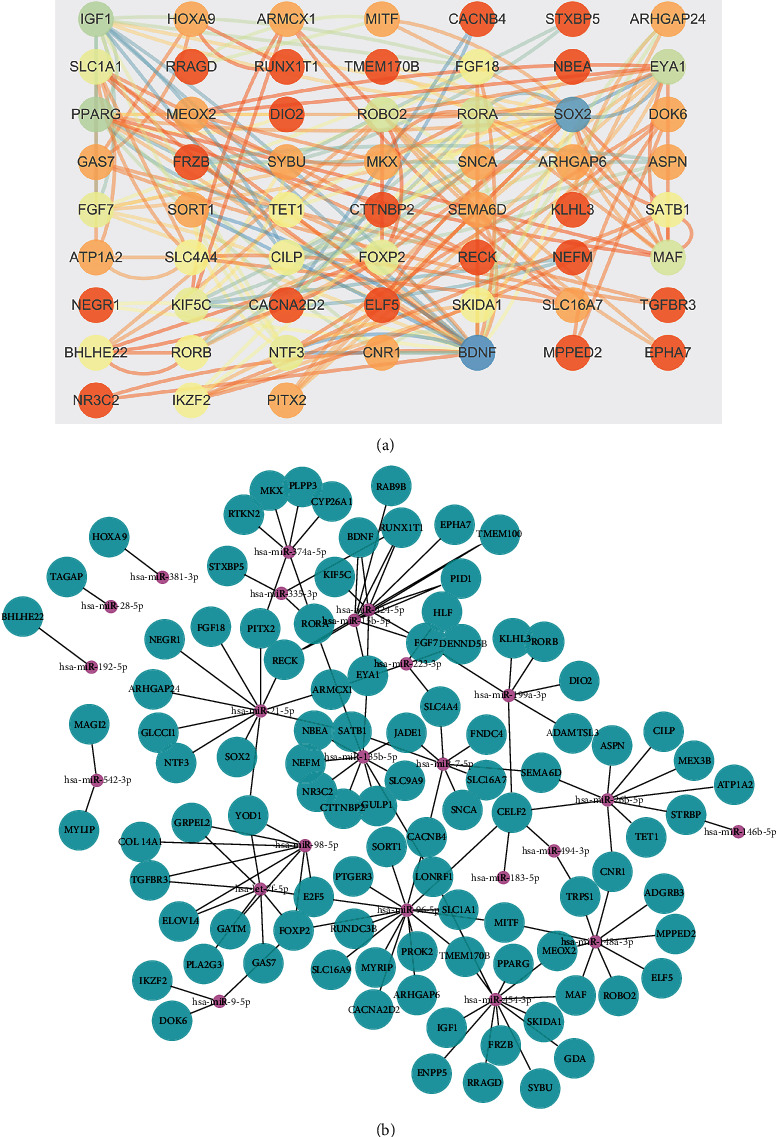
The miRNA-mRNA network related to oral cancer. (a) The PPI network of the common genes in GES28100 and GES23558. (b) The miRNA-mRNA network.

For the targets of GES28100, three clusters were found, containing cluster 1 with 31 nodes and 724 edges, cluster 2 with 54 nodes and 828 edges, and cluster 3 with 65 nodes and 504 edges (Figures 4(a)–4(c)). Three clusters were found in GSE23558, containing cluster 1 with 52 nodes and 1222 edges, cluster 2 with 53 nodes and 808 edges, and cluster 3 with 44 nodes and 566 edges (Figures 4(d) and 4(e)). Moreover, a cluster with 8 nodes and 48 edges was found in the common genes of the DEGs in GSE23558 and the targets of the DEGs in GSE28100; the miRNA-mRNA network was established (Figures 5(a) and 5(b)).

## 4. Discussion

Oral cancer is still a stubborn malignant disease with complicated pathological mechanism. The expression disorder of the genes has been gradually recognized as the major reason leading the malignant progression of the cancer [10]. This study investigated the potential regulation mechanism of oral cancer via excavating the public database and thus found some biomarker genes and the related regulation network.

Compared with the normal tissues, the miRNA profiles of the tumor tissues generally exhibited significant change [13]. Accumulating researches have indicated that some miRNAs serve critical roles in the deterioration of the tumors [14]. In the research, it was also found that the miRNA profiles were dramatically difference in the tumor tissues and adjacent healthy tissues, and 7 downregulated genes and 28 upregulated were identified as the DEG miRNAs which may serve as critical roles in oral cancer. Moreover, miR-15b-5p, miR-199a-3p, miR-21-5p, miR-424-5p, and miR-454-3p were related with the hub nodes screened in GSE23558 and the targets in GES28100. miR-199a-3p has been confirmed to drive the development of multiple types of cancer. Song et al. has observed the elevated miR-199a-3p in gastric cancer, and miR-199a-3p silence can obviously force the apoptosis of the cancer cells [15]. The research has mirrored that miR-21-5p is dramatically enriched in the exosome secreted from cancer cells which can induce the angiogenesis of tumor and thus induced the focus metastasis [16]. The research has proved that miR-15b-5p is dramatically elevated in colorectal cancer and reduced miR-15b-5p can remarkably impede the malignant behaviors of the cancer cells [17]. In oral cancer, increased miR-424-5p has been confirmed to induce the phosphorylation of STAT5 and thus drive the cellular aberrant invasion [18]. The report has indicated that miR-454-3p can induce the metastasis of breast cancer via inducing the aberrant activation of Wnt pathway [19]. However, the related research has also revealed that miR-454-3p serves as a tumor suppressor in glioma [20].

In this research, the DEGs in GSE23558, including SMAD3, PTGS2, PPARG, MMP3, THBS1, and SERPINE1, were identified as the hub nodes. Being regulated by the TGF-*β* pathway, SMAD3 has been widely proved to be involved in the deterioration of multiple tumors. Shinriki et al. have observed that the abnormal phosphorylation of SMAD3 is dramatically elevated in oral cancer cells [21]. As well as other types of cancer, oral cancer also has high invasive and migration abilities. Aberrant abundance of MMP3 is a general event which has been observed in multiple tumors, and high abundance of MMP3 is also associated with malignant phenotype of tumor cells such as invasion and migration. The research has indicated that increased MMP3 can extremely drive the metastasis of esophageal cancer cells [22]. Besides, reduced SERPINE1 can obviously impede the resistance of breast cancer cells to paclitaxel via inducing the inactivation of VEGFA [23]. In oral cancer, SERPINE1 can also induce the aberrant proliferation of the cancer cells [24]. THBS1 serves as a critical role in the angiogenesis of cancer cells and thus promotes the metastasis of oral cancer cells [25, 26]. Hence, it suggests that SMAD3, MMP3, SERPINE1, and THBS1 serve as tumor promoters in oral cancer.

Abundant researches have revealed that the aberrant changes in the activities of cellular pathways are major reasons leading the deterioration of tumor focus. The disturbances of the pathways, such as PI3K/AKT, P53, and Wnt, can directly force the malignant behaviors of tumor cells and thus have been recognized as the potential intervention target in clinical. In this research, the DEGs in both GSE23558 and GES28100 were observed to be related with the PI3K/AKT pathway, MAPK pathway, and so on. Moreover, the DEGs in GSE23558 and GES28100 were also enriched in PI3K/AKT and MAPK pathway. In oral cancer, targeting PI3K/AKT has also been recognized as a promising way in drug development. Wei et al. has found that salvianolic acid B can impede the glycolysis of oral cancer via blocking PI3K/AKT [27]. Besides, aberrant-activated MAPK pathway is also a biomarker event in the deterioration of oral cancer. Chen et al. has proved that the dysfunction of MAPK pathway can induce the TGF-*β* pathway activation and thus mediate the malignant behaviors of oral cancer cells.

In conclusion, this research identified miR-15b-5p, miR-199a-3p, miR-21-5p, miR-424-5p, and miR-454-3p as the biomarker of oral cancer and established the miRNA-mRNA network in oral progression.

## Data Availability

Data to support the findings of this study is available on reasonable request from the corresponding author.
